# Robotic versus laparoscopic gastrectomy for gastric cancer in patients with obesity: systematic review and meta-analysis

**DOI:** 10.3389/fonc.2023.1158804

**Published:** 2023-05-19

**Authors:** Xianzhe Yu, Lingling Zhu, Yan Zhang, Qingbo Feng

**Affiliations:** ^1^ Department of Gastrointestinal Surgery, Chengdu Second People’s Hospital, Chengdu, Sichuan, China; ^2^ Lung Cancer Center, West China Hospital, Sichuan University, Chengdu, Sichuan, China; ^3^ Department of General Surgery, Affiliated Hospital of Zunyi Medical University, Affiliated Digestive Hospital of Zunyi Medical University, Zunyi, China

**Keywords:** gastric cancer, robotic gastrectomy, laparoscopic gastrectomy, obesity, short-term outcomes, meta-analysis

## Abstract

**Introduction:**

The number of overweight patients with gastric cancer (GC) is increasing, and no previous study has compared laparoscopic gastrectomy (LG) and robotic gastrectomy (RG) in obese patients with GC. To investigate the perioperative and oncologic outcomes of RG and LG in obese GC patients, we performed a meta-analysis of propensity matched scores and retrospective studies to compare the perioperative parameters, oncologic findings, and short-term postoperative outcomes between the two groups.

**Methods:**

This study was performed according to the PRISMA guidelines. A search was performed on PubMed, Web of Science, EMBASE, and Cochrane Central Register to identify eligible propensity matched scores and retrospective studies conducted and published before December 2022. Data on perioperative and oncological outcomes were included in the meta-analysis.

**Results:**

Overall, we identified 1 propensity score match study and 5 randomized control trials of RG and LG, enrolling a total of 718 patients (197 and 521 patients received RG and LG, respectively). No significant differences were observed between the two groups in terms of complications, bleeding, or lymph node dissection. Of note, RG had a longer procedure time (P = 0.03), earlier oral intake (P = 0.0010), shorter hospital stay (P = 0.0002), and shorter time to defecation (P < 0.00001).

**Conclusions:**

This meta-analysis concluded that patients in the RG group had shorter hospital stays, earlier postoperative feeding, and earlier postoperative ventilation; however, no differences were found in blood loss, number of lymph nodes removed, or overall complications. RG is an effective, safe, and promising treatment for obese patients with GC, compensating for the shortcomings of laparoscopy and allowing for less trauma and faster recovery.

**Systematic review registration:**

https://www.crd.york.ac.uk/PROSPERO/, identifier CRD42022298967.

## 1 Introduction

Gastric cancer (GC) is a disease of global concern. With more than 1 million new cases of GC annually, GC is the fifth most commonly diagnosed malignancy and third most common cause of cancer-related deaths worldwide (1). GC is a complexand heterogeneous disease, resulting from the interaction among genetic, environmental, and host factors (2). Recent summaries and epidemiological studies conducted by the International Agency for Research on Cancer have reiterated obesity as a risk factor for GC (3), with the strength of the positive association between excess weight and GC risk increasing as a function of increasing body mass index (BMI) (4). Since 1980, the number of obese patients has tripled in 70 countries (5), with obesity having become a global epidemic, affecting more than 600 million adults worldwide (6). The Japanese Association for the Study of Obesity defines obesity as a BMI ≥25 kg/m^2^ (2, 7), with the criteria of the World Health Organization (WHO) differentiating between overweight (BMI between 25-30 kg/m^2^) and obesity (BMI >30 kg/m^2^) (8).

For patients with GC, radical gastrectomy with D2 lymph node dissection remains the standard treatment (9). However, minimally invasive surgery (MIS), including laparoscopic and robotic approaches, has become an effective option for the treatment of GC, particularly in patients with early-stage tumors (10, 11). Compared to conventional open gastrectomy, laparoscopic gastrectomy (LG) offers the advantages of minimal invasiveness, which include good visualization and magnification of the anatomy; less surgical trauma and pain; less intraoperative blood loss; and earlier postoperative recovery (12, 13). Since the first successful LG was reported by Kitano et al. (14) in 1994, LG has routinely been used worldwide to treat GC (15). However, obesity is considered as a major technical limitation of laparoscopic surgery, with a large amount of visceral fat narrowing the surgical field (16). Moreover, the volume and fragility of abdominal fat can also hinder pancreatic tissue and fat deposition and cause error in the intraoperative anatomical plane, resulting in inadequate lymph node dissection, which can be further exacerbated by lymph node clearance tissue and blood exudate (17).

In order to address the limitations of conventional LG in obese patients, the feasibility of robot-assisted gastrectomy (RG) has inferred from the results of earlier publications (17). Robotic surgery systems were created to overcome the effect of surgeons’ physiological hand tremor and provide a larger and more accurate range of motion, as well as to provide surgeons with a 3-dimensional (3D) magnified view of the surgical field and an ergonomic surgical environment (18). Since first reported by Hashizume et al. (19) in 2002, studies on RG have been increasingly reported. However, differences in performance parameters and surgical outcomes between robotic and laparoscopic surgery for GC have not been evaluated in obese patients. The use of MIS in this high-risk group could be improved by quality evidence regarding the risk and benefits of RG in obese patients with GC, an important clinical issue considering the rapid increase in incidence of both obesity and GC. Therefore, our aim was to perform a meta-analysis to compare perioperative parameters, oncologic findings, and short-term postoperative outcomes between RG and LG performed in obese patients with GC.

## 2 Methods

### 2.1 Search strategy and study selection

The protocol for this study was prospectively registered with PROSPERO (registration number CRD42022298967) (20) and the methods adhered to the PRISMA guidelines. The PICO model was used to ensure completeness and accuracy of the search strategy. The population of interest was obese patients with GC. The interventions evaluated were RG and LG, with outcomes compared between the two procedures or a control group. The following outcomes were evaluated: operative time, volume of blood loss, extent of lymph node dissection, overall rate of complications, time to first flatus, time to oral intake, and length of hospital stay.

A systematic literature search for published propensity score match (PSM) studies and randomized control trials (RCTs) was performed in PubMed, EMBASE, Web of Science, and Cochrane Central Register, from January 2003 to December 2022. The following keywords were used for the search: gastric cancer, obesity, laparoscopic gastrectomy, robotic gastrectomy, propensity score matching, retrospective studies, and minimally invasive surgery. A manual search of the reference lists of included studies was performed to identify additional relevant references.

### 2.2 Selection criteria and exclusion criteria

Two reviewers (XY and LZ) performed an independent screening of identified studies. In the first stage, the abstracts and titles were screened to include potentially relevant studies. Full texts of selected studies were subsequently retrieved and reviewed entirely to confirm inclusion. Disagreements were resolved by consensus with a third author (QF). The selection criteria for studies were as follows: (1) participants – mean age >18 years, confirmed diagnosis of GC, and obesity, defined by a BMI ≥25 kg/m^2^; (2) types of interventions – RG and LG; (3) types of studies – PSM and retrospective studies; and (4) inclusion of necessary data for statistical analysis or at least one of the following clinical outcomes – estimated blood loss, time to flatus, time to oral intake, number of harvested lymph nodes, operative time, length of hospital stay, and overall rate of complications. The exclusion criteria were as follows: (1) absence of differentiation between patients with GC and other indications for surgery; (2) reported on open gastrectomy only; (3) combined reporting of outcomes for LG and RG; and (4) non-comparative study designs, such as letters, reviews, comments, posters, and agreements. Observational studies in which patients were sampled based on exposure were included as cohort studies, whereas those in which patients were sampled based partially or entirely on outcomes were included as case series, irrespective of the sample size.

### 2.3 Data extraction and quality assessment

XY imported the search results into the document manager EndNoteX9. After eliminating duplicate documents, XY and LZ screened the documents by browsing titles, abstracts, and full-text reading, and XY and LZ independently extracted data from the literature that met the inclusion and exclusion criteria. The major data extraction included the following: name of first or corresponding author, study design, publication year, country, sample size, mean age, sex, BMI, operative time, bleeding, overall complications, number of retrieved lymph nodes, time to first flatus, time to oral intake, and hospital length of stay. The Newcastle-Ottawa Scale (NOS) was used to assess the quality of studies (21). All included studies were independently evaluated by two authors (XY and LZ), and RCTs and PSM studies with an NOS score >6 were considered to be high-quality studies.

### 2.4 Statistical analysis

Meta-analyses were performed using Review Manager (version 5.3). The 95% confidence interval (CI) and mean difference (MD) were used for continuous data, whereas the odds ratio (OR) with 95% CI were used for dichotomous data. When outcomes were reported as a median and range, the methods described by Hozo et al. (22) were used to convert the values to the mean and standard deviation. Statistical heterogeneity was quantified using Higgin’s I^2^ index. Larger values of I^2^ indicate increasing heterogeneity, with values of 25%, 50%, and 75% reflecting low, moderate, and high degrees of heterogeneity, respectively. If the analysis still showed significant heterogeneity, the random effects model was used for meta-analysis. A sensitivity analysis was performed by omitting one study at a time and recalculating the combined OR. Finally, Egger’s test and Begg’s method were used to evaluate bias.

## 3 Results

### 3.1 Characteristics of the included studies

**Figure 1:**
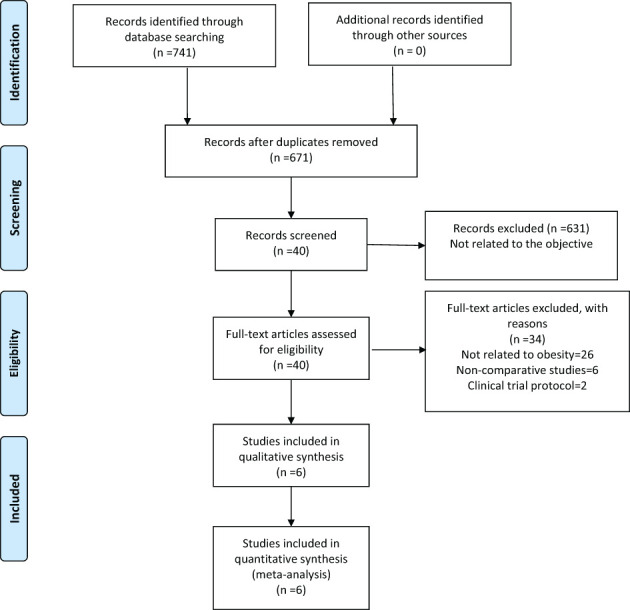
Flow chart of identification and selection of studies.

Our search retrieved 741 publications, of which six were included in the meta-analysis, one PSM (23) and five retrospective (24–28) studies, published between 2012 and 2018, reporting on a total of 718 patients. Of these, 197 patients were treated with RG for GC and 521 with LG. The detailed flow chart of the identification, screening, and selection of studies is shown in **Figure 1**. The characteristics and demographic data of patients included, as well as the summary of NOS of all included studies, are summarized in **Table 1**. The six studies included were of relatively high quality, according to the NOS (7-8) (**Table 2**).

**Table 1:** Main characteristics and NOS scores of the studies included in the meta-analysis.

Author-year	Country	RG(n)	LG(n)	Study type	Period	RG-AGE	LG-AGE	M/F RG	M/F LG	RG-BMI	LG-BMI	NOS
Hyun-2012 (24)	Korea	13	29	Retrospective	2009-2010	54.2±12.7	60.3±12.3	NA	NA	>25	>25	8
Lee-2015 (26)	Korea	31	89	Retrospective	2003-2010	58.1±11.4	62.0±10.2	26/5	56/33	26.9±1.8	26.8±1.9	7
Liu-2018 (27)	China	16	29	Retrospective	2017-2017	64 (53-67)	63 (55-68)	15/1	23/6	>30	>30	8
Li-2018 (23)	China	40	44	PSM	2013-2017	53.4±11.2	53.6±10.8	28/12	30/14	>25	>25	7
Park-2015 (25)	Korea	43	268	Retrospective	2009-2011	57.7±11.0	62.3±10.2	32/11	188/80	26.9±3.0	25.6±2.7	8
Choi-2021 (28)	Korea	54	62	Retrospective	2010-2018	59.5±12.5	63.0±14.5	40/14	45/17	26.5±2.8	26.6±2.5	8

RG, robotic gastrectomy; LG, laparoscopic gastrectomy; M/F, male/female ratio; PSM, propensity score matching; RCT, randomized controlled trial; NOS, Newcastle-Ottawa Scale; NA, not available.

**Table 2:** Study quality of included studies based on the Newcastle-Ottawa scale.

Study	Selection				Comparability	Exposure			Scores
	Adequate definition of cases	Representat- iveness of the cases	Selection of controls	Definition of controls	Control for Important factor	Ascertain- ment of exposure	Same method of ascertain- ment for cases and controls	No n- res po ns e rate	
Hyun-2012	★	★	★	★	★★	★	★	–	8
Lee-2015	★	★	★	★	★	★	★	–	7
Liu-2018	★	★	★	★	★★	★	★	–	8
Li-2018	★	★	★	★	★	★	★	–	7
Park-2015	★	★	★	★	★★	★	★	–	8
Choi-2021	★	★	★	★	★★	★	★	–	8

"★" and "★★" mean 1 and 2 points respectively.

### 3.2 Short-term outcomes

#### 3.2.1 Operative time

**Figure 2:**
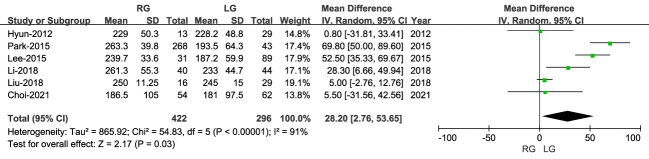
Forest plot comparing the operative time for the RG versus LG group.

The operative time was reported in six studies, with a longer operative time for RG than LG (MD: 28.20 min; 95% CI: 2.76 to 53.65; P < 0.00001), with high heterogeneity (I^2 = ^91%) (**Figure 2**).

#### 3.2.2 Blood loss

**Figure 3:**
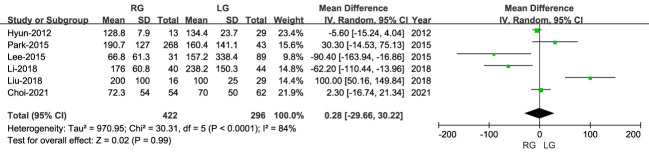
Forest plot comparing the volume of blood loss for the RG versus LG group.

The volume of blood loss was reported in six studies, including 718 patients, with lower blood loss for RG than LG (MD: 0.28 ml; 95% CI: -29.66 to 30.22; P = 0.99), with high heterogeneity (I^2 = ^84%) (**Figure 3**).

#### 3.2.3 Number of retrieved lymph nodes

**Figure 4:**
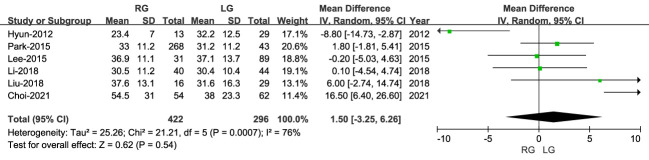
Forest plot comparing the lymph node dissection for the RG versus LG group.

The number of harvested lymph nodes was reported in six studies, with the extent of lymph node dissection not being different between RG and LG (MD: 1.50; 95% CI: -3.25 to 6.26; P = 0.54), with high heterogeneity (I^2 = ^76%) (**Figure 4**).

### 3.3 Postoperative outcomes

#### 3.3.1 Overall complications

**Figure 5:**
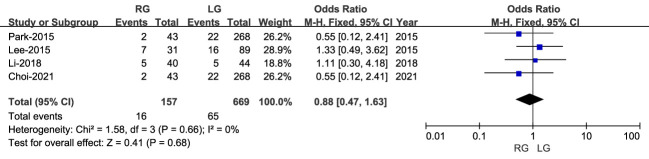
Forest plot comparing overall rate of complications for the RG versus LG group.

Overall surgical complications were reported in four studies, including 631 patients (168 RG and 463 LG). Although the rate of overall complications was lower for RG than LG, this difference was not statistically significant (OR: 0.99; 95% CI: 0.55 to 1.79; P = 0.98), with low heterogeneity (I^2 = ^0%) (**Figure 5**).

#### 3.3.2 Time to first flatus

**Figure 6:**
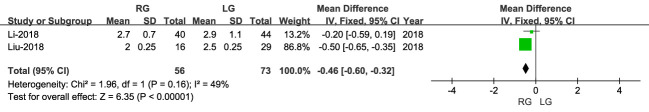
Forest plot comparing time to first flatus for the RG versus LG group.

Time to first flatus was reported in two studies, including 129 patients (56 RG and 73 LG), and was lower for RG than LG (OR: -0. 46; 95% CI: -0.60 to -0.32; P < 0.00001), with moderate heterogeneity (I^2 = ^49%) (**Figure 6**).

#### 3.3.3 Time to oral intake

**Figure 7:**
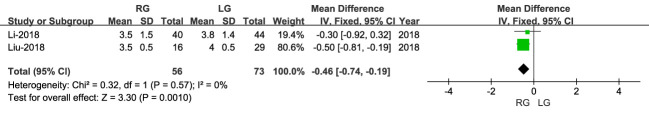
Forest plots comparing the time to oral intake for the RG versus LG group.

Time to oral intake was reported in two studies, including 129 patients (56 RG and 73 LG). The time to oral intake was earlier for RG than LG (OR: -0.46; 95% CI: -0.74, -0.19; P = 0.0010), with low heterogeneity (I^2 = ^0%) (**Figure 7**).

#### 3.3.4 Length of hospital stay

**Figure 8:**
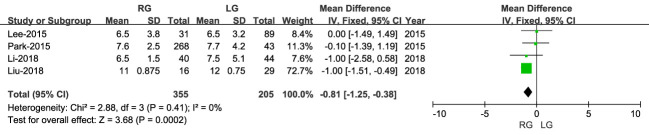
Forest plots comparing the length of hospital stay for the RG versus LG group.

**Figure 9:**
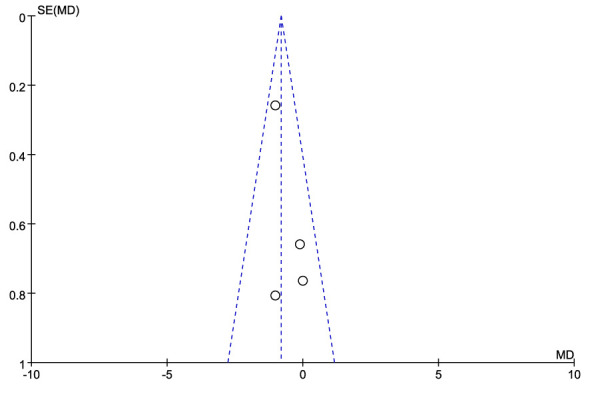
Funnel plots comparing length of hospital stay for the RG versus LG group.

Length of hospital stay was reported in four studies, including 560 patients (355 RG and 205 LG), with a shorter stay for RG than LG (MD: - 0.81; 95% CI: -1.25 to 0.38; P = 0.0002), with low heterogeneity (I^2 = ^0%) (**Figures 8**, **9**).

## 4 Discussion

MIS has revolutionized gastrectomy surgery, with comparable oncological outcomes to those for open surgery having been reported, making it an ideal alternative to open surgery to facilitate an earlier return to daily activities postoperatively (29). MIS offers many advantages over conventional open surgery, including reduced post-operative pain, rapid recovery of gastrointestinal function, and shorter hospital stays (30). In their meta-analysis study, Milone et al. (31) showed that for distal gastrectomy, a total laparoscopic approach, with intraperitoneal anastomosis, was safe and feasible, compared to laparoscopic-assisted surgery with extraperitoneal anastomosis, with a lower volume of blood loss (P=0.003), lower number of lymph nodes harvested (P=0.022), and shorter length of hospital stay (P=0.037). However, differences between RG an LG have not previously beenclarified for obese patients with GC with regards to perioperativeparameters, oncological findings, and short-term postoperative outcomes.

The increasing prevalence of obesity and overweight is now considered to be a global epidemic, with 1.9 billion and 650 million of adults being obese and overweight, respectively, in 2016 (32). The International Obesity Task Force recommended that a BMI ≥25 kg/m^2^ be used to define obesity (33). Obesity poses a specific risk for radical laparoscopy for GC treatment. While radical laparoscopic treatment for GC offers significant advantages over conventional open surgery in terms of minimizing invasive operative time and shortening the postoperative recovery period (34), the approach is limited by two-dimensional images, decreased tactile sensation, magnification of physiological hand tremor, decreased dexterity due to instruments used, and limited range of instrument motion (35). The large amount of fatty tissue in the abdominal wall and cavity in obese patients increases the difficulty of adequately exposing the surgical field, further increasing the technical difficulty of radical laparoscopic treatment (36). This increased technical difficulty has been associated with longer operative times, increased intra-operative blood loss, decreased extent of lymph node dissection, and a higher risk of postoperative complications (37, 38). Major complications of distal gastrectomy for GC treatment in obese patients include pancreatic fistula and significant increase in anastomotic leakage (39). Robotic surgery has been shown to overcome the limitations associated with a thick abdominal wall and excess intra-abdominal fat for colectomy.

Therefore, new technology is required to overcome these limitations. For patients who have undergone colectomy (40), robotic surgery has been shown to overcome the difficulties associated with thick abdominal walls and excess intra-abdominal fat, improving the visual field, instrumentation, and ergonomics. Therefore, the benefits of robotic surgery might be more pronounced for patients with a high BMI compared to those with BMI within normal range. RG improves complex reconstruction after gastrectomy and lymph node dissection to ensure oncological safety in patients with advanced GC (15), providing an enlarged 3D view and steadily motion of tweezers, allowing for precise identification of anatomical layers and avoiding injury to the adjacent organs (41). In particular, wrist mounted surgical instruments combined with the shock absorption function of RG can provide accurate anatomical rendering near the pancreas for precise *in vivo* anastomosis, which can reduce postoperative intraperitoneal complications (42). These features minimize surgical trauma and facilitate precise control of intra-abdominal bleeding in technically challenging tasks, such as lymph node dissection, intracorporeal anastomosis, and application of ligatures within closed cavities (43). As surgical instruments continue to advance and surgical performance gradually improve, more procedures have been attempted by experienced surgeons using Da Vinci robots, with the safety and feasibility of these procedures having been confirmed (44). Moreover, the learning curve for RG is less steep than that for LG (45), with better outcomes obtained in initial cases for RG than LG, indicative of an easier adaptation to robot-assisted than laparoscopic surgery. However, no previous studies have reported on the advantages of RG over LG with regards to reducing surgical stress and improving short-term outcomes in patients with obesity.

Longer operative time for RG than LG might be attributed to the longer docking time associated with older robotic systems, which often involve complex docking procedures and port-placement configurations (25). Longer operative time for RG in obese patients include unsuitable length of ports for obese patients, inefficient port placement, camera movements that interrupt the procedure, and unsuitable optical systems that do not allow for a large surgical field of view, all of which prevent safe continuous dissection and require careful manipulation (46). However, with the newer Da Vinci Xi system, docking can be accomplished more quickly, using an arm-mounted robotic arm and a laser targeting system. In addition, the multi-quadrant capability of Xi system reduces the need for redocking or mixing procedures, thereby reducing the procedure time (41). Song et al. (47) reported a decrease in time to dock and set up of the robotic arm after the initial 25 learning cases, with further shortening of docking time with accumulating experience. The effect of learning curve may have contributed to the longer operative time in the RG group as some cases were performed as a part of the surgeon’s learning. This is a limitation of the evidence we present in this study and, therefore, the prolonged operation time should not limit the research on new applications of RG.

Radical surgery for GC requires extensive lymph node dissection for accurate assessment of GC stage and prognosis, reducing the risk for metastasis and recurrence (48, 49). In our analysis, there was no difference in the extent of lymph node dissection between RG and LG (P = 0.82). Moreover, surgical blood loss (P = 0.99) and rate of complications (P = 0.98) were also comparable between the two approaches. However, RG offered surgeons the benefit of a 3D surgical field of view, with a magnification of 10-15x, improving direct viewing of the relationship between blood vessels and surrounding tissues and ability to clearly recognize different tissue structures. In addition, the manipulator arm (e.g., “hand” of the robotic surgical system) removes physiological tremor, improving surgical stability and accuracy, thus ensuring safety during gastric vascular dissection and ligation (50). In this regard, the rate of postoperative complication is an important short-term outcome, being lower for RG than LG group, although this difference was not statistically significant. These results demonstrate that RG is as safe and as viable as LG for the treatment of GC in obese patients.

Since the first description of RG in 2003, its use has increased rapidly, especially in East Asia (51). Our analysis identified two important advantages of RG over LG, namely: a faster return to bowel function and shorter length of hospital stay. Earlier time to first flatus and time to first oral intake are two crucial factors of postoperative recovery. The superiority of RG over LG on functional recovery of bowel function may reflect the stability and flexibility of the movement of the robotic arm, avoiding excessive traction forces on the tissues and accidental damage to the blood vessels, and, thus, causing less trauma to the patient (52). Therefore, RG causes minimal disturbance to the gastrointestinal tract, facilitating an earlier recovery of bowel function, resulting in earlier return to oral intake, evacuation, and early discharge from hospital. However, few studies have evaluated the long-term oncological efficacy of RG, with most of these having limitations, such as imbalanced covariates, a small number of cases, and the inability to compare outcomes with the same surgical team (53). Moreover, owing to the short history of clinical use of RG, the long-term survival outcomes between RG compared to other surgical methods for GC in obese patients remains to be evaluated.

Owing to insufficient data, the cost-effectiveness of RG and LG in obese patients with GC was not compared in this meta-analysis. Robotic surgical systems have well-known capital and maintenance costs, as well as additional costs depending on the robot-assisted procedures (54). However, there is growing evidence that robotic surgery is more cost effective in the long term, compared to traditional open surgery, reducing the length of hospital stay and lowering the risk of complications (55). Robotic surgery might have specific cost benefits for high-risk clinical populations, such as the elderly, morbidly obese patients, and patients with comorbidities (56). Moreover, the cost of robotic gastrectomy surgery will gradually decrease with continued improvement in robotic technology and increased use.

In summary, our meta-analysis provides evidence of specific benefits of RG over LG for GC treatment in obese patients, namely shorter hospital stay, and earlier oral intake and bowel function. Therefore, RG might be considered as an effective, safe, and promising treatment for obese patients with GC, resulting in less trauma than LG and facilitating a faster postoperative recovery. Long-term oncological outcomes and survival of RG will need to be evaluated. It is anticipated that future prospective trials with long-term outcomes will provide a better understanding of the role of RG in the treatment of GC in obese patients.

## Data availability statement

The original contributions presented in the study are included in the article/supplementary material, further inquiries can be directed to the corresponding author/s.

## Author contributions

Study concept and design (XY and LZ). Acquisition of data (QF). Analysis and interpretation of data (QF). Drafting of the manuscript (XY and LZ). Critical revision of the manuscript for important intellectual content (YZ and LZ). Administrative, technical, or material support; and study supervision (YZ and QF). All authors have contributed to the manuscript and approved the submitted version.
